# Cardiac Magnetic Resonance to Reclassify Diagnosis and Detect Cardiomyopathies in Hospitalized Patients with Acute Presentation

**DOI:** 10.3390/life15030470

**Published:** 2025-03-15

**Authors:** Theodoros Tsampras, Alexios Antonopoulos, Alexandros Kasiakogias, Alexia Mika, Antonia Kolovou, Eleni Papadimitriou, George Lazaros, Konstantinos Tsioufis, Charalambos Vlachopoulos

**Affiliations:** 1 1st Cardiology Department, Hippokration Hospital, National and Kapodistrian University of Athens, 11527 Athens, Greece; teodore.tsampras@gmail.com (T.T.); akasiakogias@gmail.com (A.K.); glaz35@hotmail.com (G.L.); ktsioufis@gmail.com (K.T.); cvlachop@otenet.gr (C.V.); 2 Radiology Department, Hippokration Hospital, 11527 Athens, Greece

**Keywords:** cardiomyopathy, cardiovascular magnetic resonance, heart failure, myocarditis, myocardial infarction

## Abstract

Background: Cardiomyopathies are a significant cause of heart failure, arrhythmia, and cardiac morbidity in the general population. Cardiovascular magnetic resonance (CMR) is a valuable tool for the diagnostic work-up of patients with acute cardiac events. Objectives: This study evaluated the diagnostic value of CMR and the yield of cardiomyopathies in hospitalized cardiac patients with acute presentation. Methods: A retrospective analysis was conducted with 535 consecutive hospitalized patients who underwent CMR at Hippokration Hospital, Athens, Greece, to identify a subset of scans performed on an urgent basis of hospitalized patients. Demographic data, causes of admission, CMR findings, and plasma cardiac biomarkers (hs-Troponin I, NT-proBNP, and CRP) were systematically recorded. Results: Out of the initial 535 CMR scans evaluated, a further analysis was conducted with 104 patients who were in hospital and underwent CMR on an urgent basis. From the total population of hospitalized patients, 33% had CMR findings indicative of underlying cardiomyopathy, with dilated cardiomyopathy being the most common subtype (36%), followed by arrhythmogenic cardiomyopathy (27%), hypertrophic cardiomyopathy (15%), or other subtypes (e.g., cardiac amyloidosis, sarcoidosis, endomyocardial fibrosis, EGPA, or unclassified). CMR led to the reclassification of the initial diagnosis into that of underlying cardiomyopathy in 32% of cases. The highest reclassification rate was observed within the subgroup with heart failure (71%), followed by that of acute myocardial infarction/ischemic heart disease (24%) and myocarditis (22%). Conclusions: CMR imaging effectively contributed to the differential diagnosis of hospitalized patients with acute cardiac events that remained without a definitive diagnosis after their initial work-up and uncovered underlying cardiomyopathy in almost one-third of this cohort.

## 1. Introduction

Cardiomyopathies are a significant cause of morbidity and mortality [1] and among the major causes of heart failure and sudden cardiac death in young individuals [2]. Risk stratification and prognosis is linked with the underlying cardiomyopathy subtype, and accurate diagnosis is essential for optimal treatment and patient management [3,4]. Cardiomyopathies may lead not only to symptoms of chronic heart failure (HF) [5] but also to acute symptoms including chest pain, new-onset ventricular arrhythmias (VAs), or the decompensation of HF [6,7,8]. Although once considered rare, cardiomyopathies collectively account for a significant number of patients presenting with acute cardiac symptomatology [9]; increased vigilance is crucial to prevent misdiagnosis.

Cardiovascular magnetic resonance (CMR) is a valuable noninvasive diagnostic technique for assessing cardiomyopathy phenotypes [10,11,12]. CMR not only provides information on biventricular volumes and systolic function but also offers characterization of myocardial tissue, aiding in the differential diagnosis [13,14]. Multiparametric CMR imaging enables the detection of fibrotic tissue and myocardial edema [15,16,17], or even myocardial infiltration with amyloid, iron, or fat [13]. This is crucial for raising the suspicion of rare cardiomyopathies that often remain undetected [18,19]. The value of CMR in diagnosing cardiomyopathies is highlighted in the recent relevant clinical recommendations for the management of patients with cardiomyopathy, where CMR has a class I indication for all cardiomyopathy patients at initial evaluation [20].

In the acute setting, CMR plays a role in the differential diagnosis of patients with myocardial infarction with non-obstructive coronary arteries (MINOCA) [21,22]. It is estimated that CMR can lead to reclassification and determine the specific diagnosis in approximately 70% of such cases [23]. CMR contributes to the etiological diagnosis of patients with acute HF [24] and in patients with VAs to assess for the presence of arrhythmic substrate, i.e., scarring, which may be associated with their clinical arrhythmias [25]. Therapeutic interventions can, therefore, also be guided by CMR findings.

Since the diagnostic yield of CMR imaging for cardiomyopathies in the acute hospital setting has not been systematically evaluated, we aimed to assess the value of CMR in reclassifying diagnoses and establishing a cardiomyopathy diagnosis in acutely ill hospitalized cardiac patients. These patients remained undiagnosed after the initial in-hospital work-up, posing both a diagnostic and therapeutic challenge.

## 2. Methods

### 2.1. Study Design

The inclusion criterion for this study was the performance of an in-hospital CMR scan at Hippokration Hospital, Athens, Greece, from the start of CMR clinical service implementation in January 2020 up to March 2023. The study included only hospitalized patients who underwent an urgent CMR scan, excluding outpatients. No additional inclusion criteria were applied.

Among the 535 CMR scans conducted during this period, 103 were performed on an urgent basis for hospitalized patients with acute cardiac symptomatology as part of their diagnostic algorithm. These scans were selected for further analysis. Demographic characteristics such as age, sex, and ethnicity, along with the CMR date, admission cause, diagnosis, and relevant electrocardiographic findings, were documented. Functional CMR parameters and laboratory markers, e.g., high-sensitivity Troponin I (hs-Troponin I), B-type Natriuretic Peptide (BNP)/N-terminal pro-B-type Natriuretic Peptide (NT-proBNP), and high-sensitivity C-reactive Protein (hs-CRP), were also recorded when available.

The objective of the study was to assess the diagnostic and reclassification value of CMR in acutely ill cardiac patients, with a specific focus to explore its yield for cardiomyopathies in this special population of hospitalized patients with acute clinical presentation that remained without a definitive diagnosis after their initial diagnostic work-up.

### 2.2. CMR Imaging

All CMR scans of hospitalized patients were retrospectively reviewed by Level 3 CMR experts blinded to the initial or final clinical diagnosis. Studies that were not eligible for analysis for technical reasons (i.e., missing images or bad image quality) were omitted, and the corresponding patients were excluded from the analysis (*n* = 4). The analysis sought to identify potential underlying cardiomyopathies and classify the specific type for each patient. The diagnoses were determined solely based on the findings from the CMR images, as evaluated by a Level 3 CMR specialist. No additional data from the patients’ laboratory studies or medical history were utilized to establish the final diagnosis. Reclassification was defined by comparing the initial cause of admission with the diagnosis derived from the CMR findings.

### 2.3. CVI42 Measurements

A quantitative analysis of ventricular volumes, systolic function, and myocardial mass was performed using the CVI42 v6.1 software. Standard CMR-derived parameters included left and right ventricular end-diastolic and end-systolic volumes, stroke volumes, ejection fractions, and cardiac output. Myocardial mass was assessed in both the systole and diastole. The presence of Late gadolinium enhancement (LGE) in PSIR images was also evaluated to identify the presence of myocardial fibrosis. The use of this software ensured reproducibility in the assessment of cardiac function and tissue characterization.

### 2.4. Statistical Analysis

All statistical analyses were performed using the GraphPad Prism 10.1.1 Software. The frequencies of all variables were calculated, and data were categorized accordingly. Continuous variables, due to their skewed and asymmetric distribution, are presented as medians, along with interquartile ranges (IQRs), to provide a summary of the central tendency and variability. Categorical variables are expressed as percentages, offering a depiction of the distribution across groups. Comparisons between groups were carried out using statistical tests based on data type and distribution. Non-parametric methods were employed for continuous variables due to the lack of normality, while chi-square or Fisher’s exact tests were used for categorical data. Statistical significance was defined as a *p*-value < 0.05.

## 3. Results

### 3.1. Patient Demographics and Causes of Hospital Admission

**Figure 1:**
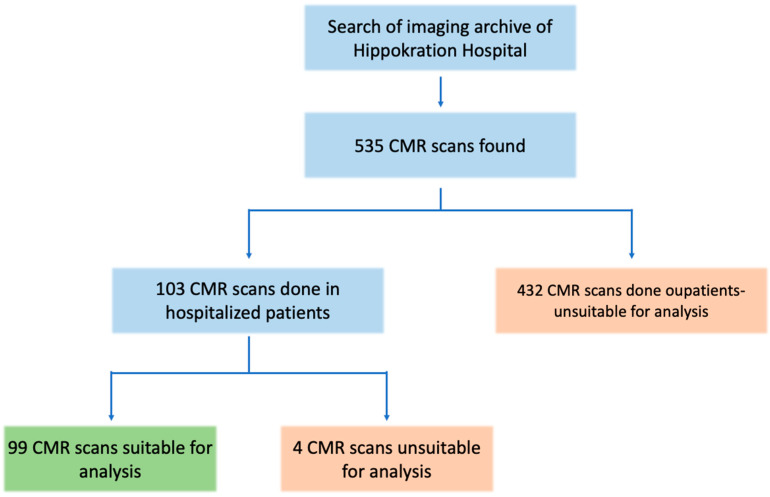
Study flowchart. Out of the initial 535 CMR scans, 99 scans were included in the analysis of hospitalized patients with acute cardiac events.

A total of 535 CMR scans were retrieved from the hospital’s archive search. Of these, 103 scans were performed with hospitalized patients. Four scans were excluded from the analysis due to the lack of gadolinium contrast or missing LGE images. The remaining 99 scans, all from unique inpatients, were included in the study for further analysis (Figure 1). The median age of this in-patient sample was 47 years (IQR: 31–63), and 35% of the patients were female. Detailed demographic information, along with data on CMR findings and biomarkers for the study population, are presented in Table 1.

**Figure 2:**
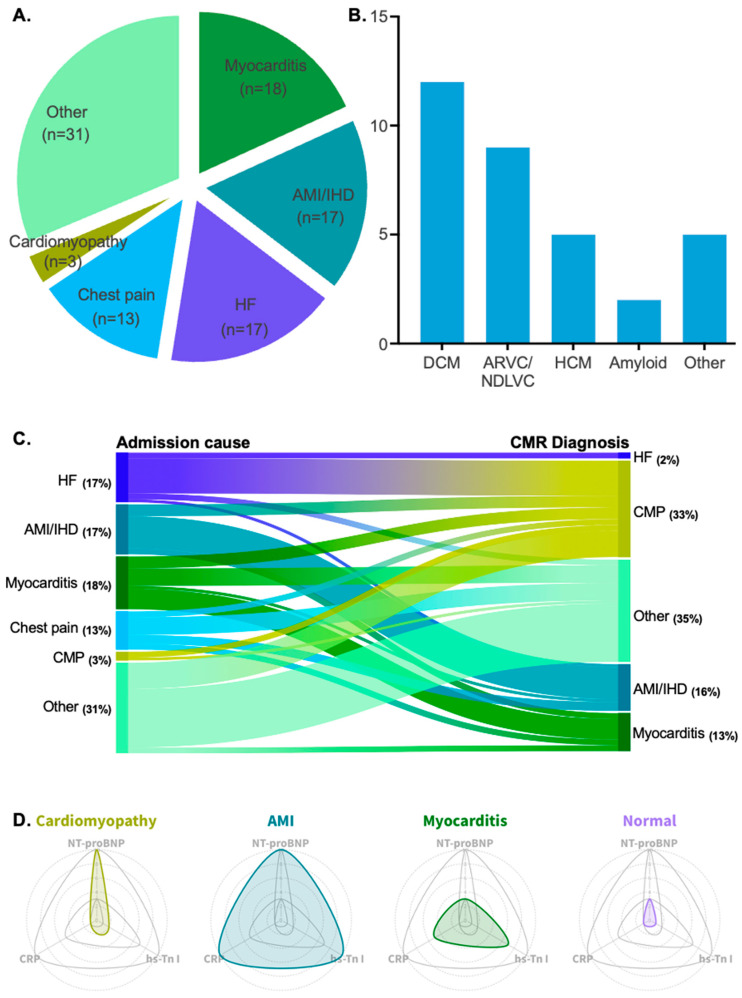
(**A**) Pie chart showing the patient cohort divided by admission cause. (**B**) Bar chart showing all diagnosed cardiomyopathy types with their corresponding patient count. (**C**) Alluvial plot showing the reclassification of diagnoses after the implementation of CMR imaging. (**D**) Radar plots showing the fluctuation of hs-Tn I, NT-proBNP, and CRP in four different patient groups: cardiomyopathy patients, AMI/IHD patients, myocarditis patients, and patients with normal CMR scans.

Among the clinical indications for hospitalization, possible myocarditis was the most common cause of admission, accounting for 18% of cases. Acute myocardial infarction (AMI)/ischemic heart disease (IHD) and heart failure HF were the second most common causes of admission, each accounting for 17%. Chest pain followed, with a frequency of 13%. Lastly, cardiomyopathy as the primary cause of admission was reported in 3% of cases. All patient admission causes are depicted in Figure 2A.

### 3.2. Clinical Diagnosis via CMR

Retrospective analysis via CMR scans revealed that 33% (*n* = 33) of patients exhibited findings indicative of underlying cardiomyopathy. Among these patients, dilated cardiomyopathy was the most prevalent clinical entity (36%, *n* = 12). Arrhythmogenic cardiomyopathy (arrhythmogenic right ventricular cardiomyopathy or left-dominant forms in the non-dilated left ventricular cardiomyopathy spectrum) and hypertrophic cardiomyopathy were the second and third most frequent types, with frequencies of 27% (*n* = 9) and 15% (*n* = 5), respectively. Other rarer forms of cardiomyopathy included cardiac sarcoidosis, eosinophilic granulomatosis with polyangiitis, and endomyocardial fibrosis. All types of diagnosed cardiomyopathies are listed in Table 2 and illustrated in Figure 2B.

Additionally, myocarditis was the most probable diagnosis in 13% (*n* = 13) of the cases, while AMI/IHD accounted for 8% (*n* = 8) of diagnoses. The absence of any abnormal CMR findings was recorded in 26% (*n* = 26) of the scans, which were considered normal scans. Lastly, 19% (*n* = 19) of the CMR scans were suggestive of other diagnoses (e.g., pericarditis, valvular heart disease, or vasculitis with cardiac involvement).

### 3.3. Reclassification of Clinical Diagnosis via CMR

Interestingly, patients from all main admission categories were significantly reclassified into different diagnostic categories after the implementation of CMR (chi^2^ = 104.1, *p* < 0.0001). In total, 32% (*n* = 31) of patients with reported admission causes of heart failure, chest pain, AMI/IHD, acute myocarditis, or other cardiac events (e.g., arrhythmia, pericarditis, or conduction disorders) were reclassified as having an acute presentation of cardiomyopathy.

**Figure 3:**
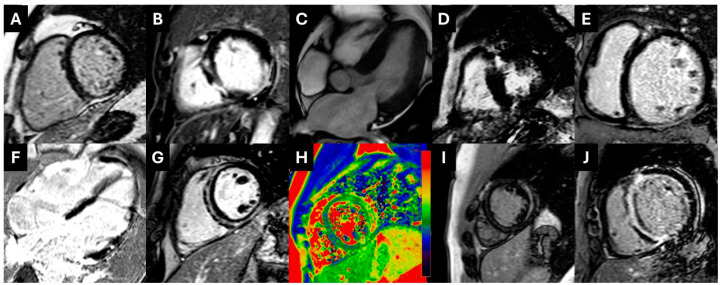
(**A**). PSIR images showing subepicardial inferior wall LGE extending to the right ventricle compatible with ARVC with biventricular involvement. (**B**). Transmural lateral wall plus midwall LGE in the interventricular septum in the setting of NDLVC. (**C**,**D**). Patient with CMR findings compatible with HOCM; symmetrical concentric LV hypertrophy (**C**) and presence of extensive LGE in the lower interventricular insertion point (**D**). (**Ε**). Full-blown DCM with midwall LGE in the IVS. (**F**). Extensive–diffuse LGE in multiple myocardial walls compatible with sarcoid cardiomyopathy. (**G**,**H**). Patient with ring-like subepicardial and midwall LGE compatible with left-dominant ACM (NDLVC) (**G**) and myocardial edema in T2-Mapping (**H**). (**I**). Subepicardial LGE in the mid-lateral wall and intramural LGE in the interventricular septum compatible with myocarditis and possible underlying cardiomyopathy. (**J**). Diffuse subendocardial to full-thickness LGE in a patient with cardiac amyloidosis. ACM: arrhythmogenic cardiomyopathy; ARVC: arrhythmogenic right ventricular cardiomyopathy; DCM: dilated cardiomyopathy; HOCM: hypertrophic obstructive cardiomyopathy; IVS: interventricular septum; NDLVC: non-dilated left ventricular cardiomyopathy; LGE: late gadolinium enhancement; LV: left ventricular.

The highest reclassification rate was observed among patients admitted for HF decompensation. In 71% (*n* = 12) of these patients, underlying probable cardiomyopathy was identified. Patients with an initial diagnosis of AMI/IHD were reclassified into a cardiomyopathy diagnosis at an estimated frequency of 24% (*n* = 4). Patients who were admitted to the hospital for a myocarditis episode were found to have underlying cardiomyopathy at a frequency of 22% (*n* = 4). Lastly, a substantial percentage of patients admitted for chest pain were reclassified after CMR towards an acute presentation of underlying cardiomyopathy (15%, *n* = 2), and an equal percentage was reclassified as having an acute myocarditis episode (15%, *n* = 2). The reclassification rate achieved via CMR is depicted in Figure 2C. Examples of CMR images with underlying non-ischemic cardiomyopathy in the studied population are depicted in Figure 3.

### 3.4. Biomarker Values Among Different Patient Groups

A distinct biomarker pattern was identified among the different patient groups based on CMR findings. Patients exhibited varying levels of hs-Troponin I, NT-proBNP, and CRP, with significant differences observed between those with cardiomyopathy, myocarditis, AMI/IHD, and normal CMR scans. Cardiomyopathy patients had significantly lower hs-Troponin I levels than those with myocarditis and AMI/IHD (*p* < 0.01), highlighting a clear differentiation based on troponin levels. NT-proBNP levels were higher in cardiomyopathy patients compared to those with myocarditis, even though the difference did not reach statistical significance (*p* = 0.21). CRP levels were also lower in cardiomyopathy patients compared to myocarditis and AMI/IHD groups, with both differences reaching statistical significance (*p* < 0.05). These biomarker variations suggest a distinct inflammatory and myocardial injury profile in cardiomyopathy. A detailed comparison of biomarker values is presented in Table 3, while Figure 2D illustrates the observed pattern and its diagnostic implications.

## 4. Discussion

The findings of the present study suggest that a significant proportion of acutely ill cardiac patients with an indication to undergo a CMR to aid in the diagnostic assessment had findings indicative of underlying cardiomyopathy. Referral for a CMR scan at an urgent in-hospital setting frequently led to a reclassification of a diagnosis among hospitalized patients with an acute cardiac presentation, and almost one-third of these patients were diagnosed with probable/definite cardiomyopathy.

Dilated cardiomyopathy was identified as the most commonly diagnosed type among cardiomyopathies, consistent with the existing literature that identifies it as the most prevalent form [26,27,28]. Interestingly, the second most commonly diagnosed cardiomyopathy in this group of patients was arrhythmogenic cardiomyopathy (right- or left-dominant forms), a disease whose prevalence in the general population is considered significantly lower [29,30]. This is especially important, as myocarditis episodes can frequently serve as the initial clinical presentation of underlying arrhythmogenic cardiomyopathy [31,32,33]. Therefore, heightened awareness regarding the early use of CMR imaging is crucial for the timely diagnosis of such patients [34,35].

Cardiac biomarkers such as hs-troponin I, NT-proBNP, and CRP are not disease-specific biomarkers, but they can aid in the diagnostic work-up of myocardial diseases [36]. In this study, a specific pattern pointing to a cardiomyopathy diagnosis was identified. Acutely ill cardiac patients with underlying cardiomyopathy exhibit significantly elevated NT-proBNP levels, accompanied by only mildly elevated hs-Troponin I and CRP levels [37]. This combination can increase the clinician’s suspicion for the existence of underlying cardiomyopathy, as the main alternative diagnoses show different elevation patterns for these three biomarkers [38,39,40].

CMR can also inform treatment decisions, thereby improving patient prognosis. In the absence of CMR implementation, patients admitted for heart failure decompensation would likely be treated symptomatically, without the underlying condition being addressed [41]. Latent cardiomyopathies would remain undiagnosed, meaning that no risk stratification for these patients would be performed, and life-saving interventions, like the implantation of an implantable cardioverter–defibrillator, would not be considered [20].

MINOCA is a heterogeneous group of diseases, making specific diagnoses challenging [42]. Definitive diagnoses of patients presenting with MINOCA are often unachievable without the use of CMR, and individualized treatment, like dual-antiplatelet therapy and angiotensin-converting enzyme inhibitors in patients with evidence of an ischemic scar/NSTEMI, as indicated through CMR, would not be initiated on time [43]. Conversely, in patients presenting with MINOCA or those initially diagnosed with myocardial infarction, CMR can reveal non-ischemic cardiac events (e.g., myocarditis or an acute presentation of cardiomyopathy). In such cases, CMR prevents unnecessary empirical treatment with anti-ischemic agents, which would provide no benefit [23]. These adjustments in medical management, along with the frequent need for additional diagnostic testing prompted by CMR findings, highlight the crucial role of the modality in managing hospitalized patients [44].

Lastly, without the implementation of CMR, patients presenting with myocarditis episodes linked to underlying cardiomyopathy may remain undiagnosed and receive treatment solely as a myocarditis episode, with a failure to address the underlying cause. Identifying cardiomyopathies through CMR can guide therapeutic interventions, thereby enhancing prognoses and reducing the risk of acute cardiac events such as sudden cardiac death or heart failure decompensation [45].

## 5. Limitations

This study involved limitations that should be considered when interpreting the results. First, it was a single-center, retrospective analysis conducted at Hippokration Hospital, Athens, Greece. This limits the generalizability of the findings to other healthcare settings and populations. The study was performed at a single tertiary care center, which may have inherent biases related to the referral patterns and patient characteristics specific to this institution. As such, the results may not fully reflect the diagnostic yield of CMR in other hospital settings, particularly in non-tertiary hospitals or community-based care environments. Furthermore, the patient cohort in this study was relatively small. While this number provides valuable insights, larger multicenter studies with a more diverse sample population are needed to confirm the findings and assess the applicability of CMR in broader patient populations. A larger cohort would also help address any statistical power issues and allow for more robust subgroup analyses. Lastly, as part of a retrospective study, the data were collected from existing medical records, which may be subject to documentation errors or incomplete information. The reliance on archived clinical data may have limited the ability to account for all relevant factors, such as variations in clinical management, patient comorbidities, or the potential influence of unmeasured variables. Additionally, patients with poor image quality or missing data were excluded from the analysis, which could have resulted in selection bias, potentially affecting the study’s findings. Lastly, electrocardiographic data were inadequately documented, and echocardiographic data were absent from the available patient records. As a result, these data were not included in the analysis.

## 6. Conclusions

In hospitalized patients with acute cardiac presentation who require urgent CMR imaging as part of their diagnostic work-up, the diagnostic yield for cardiomyopathies is substantial. The reclassification rate of CMR is significant, with almost one-third of patients with acute cardiac presentation having findings of underlying cardiomyopathy in this cohort. In-hospital CMR enables diagnostic reclassification, as well as the timely identification of underlying cardiomyopathy as the cause of acute cardiac presentation, and it informs therapeutic decisions. The setting up of a clinical CMR service for cardiology inpatients at a tertiary hospital center helps with the diagnosis of common and rarer forms of cardiomyopathy before hospital discharge.

## Figures and Tables

**Table 1:** Demographic and clinical characteristics of hospitalized patients included in the analysis.

***Demographics***	
Patients, *n*	99 (100)
Age, median, years	47 [31–63]
Male sex, *n* (%)	64 (64.7) *
Race	
Caucasian, *n* (%)	83 (83.8) *
Asian, *n* (%)	16 (16.2) *
CMR, days from admission	3 [2–6]
***CMR parameters***	
LVEDV, mL	178.4 ± 8.5
LVESV, mL	98.4 ± 8.1
LVSV, mL	80.0 ± 3.0
LVEF, %	48.5 ± 1.7
LVCO, L/min	5.6 ± 0.2
LV Myo Mass Diast, g	116.5 ± 5.3
LV Myo Mass Syst, g	121.2 ± 5.5
RVEDV, mL	169.0 ± 7.6
RVESV, mL	92.2 ± 6.5
RVSV, mL	76.8 ± 3.0
RVEF, %	47.4 ± 1.4
RVCO, L/min	5.4 ± 0.2
LGE, *n* (%)	57 (57.6)
***Hematologic tests***	
cTnI, pg/mL	173.0 [35.1–3019.0]
NT-proBNP, pg/mL	628.5 [221.2–3086.0]
CRP, mg/L	19.0 [5.7–60.1]

CMR: cardiovascular magnetic resonance; CRP: c-reactive protein; cTnI: cardiac troponin I; LVCO: left ventricular cardiac output; LVEDV: left ventricular end-diastolic volume; LVEF: left ventricular ejection fraction; LVESV: left ventricular end-systolic volume; LVSV: left ventricular stroke volume; NT-proBNP: N-terminal pro-B-type natriuretic peptide; RVCO: right ventricular cardiac output; RVEDV: right ventricular end-diastolic volume; RVEF: right ventricular ejection fraction; RVESV: right ventricular end-systolic volume; RVSV: right ventricular stroke volume. Continuous variables are reported as mean ± SEM or median [IQR], as appropriate. * *p* < 0.05.

**Table 2:** Types of cardiomyopathies diagnosed with CMR.

Type of Cardiomyopathy	N
DCM	12 (36%)
ARVC/ACM (NDLVC)	9 (27%)
HCM	5 (15%)
CA	2 (6%)
Other	5 (15%)
*Cardiac sarcoidosis*	1 (3%)
*EGPA*	1 (3%)
*Endomyocardial fibrosis*	1 (3%)
*Unclassified cardiomyopathy*	2 (6%)

ARVC: arrhythmogenic right ventricular cardiomyopathy; CA: cardiac amyloidosis; DCM: dilated cardiomyopathy; HCM: hypertrophic cardiomyopathy; NDLVC: non-dilated left ventricular cardiomyopathy; EGPA: eosinophilic granulomatosis with polyangiitis.

**Table 3:** Comparison of cardiac biomarkers among patient groups based on CMR findings.

Biomarker	Cardiomyopathy	Myocarditis	AMI/IHD	Normal CMR	*p*-Value
cTnI (pg/mL)	151.7 [63.4–1940.0]	5763.0 ** [2524.0–16,922.0]	14,157.0 ** [2762.0–48,310.0]	107.8 [28.4–339.3]	*p* < 0.0001
NT-proBNP (pg/mL)	2263.0 [216.2–4143.0]	458.8 [357.1–604.6] *	2336.0 [2083.0–9196.0]	574.0 [123.4–1823.0]	*p* = 0.10
CRP (mg/L)	10.9 [5.9–52.7]	45.8 * [25.8–85.8]	128.4 * [21.7–145.9]	8.6 [2.7–38.1]	*p* = 0.01

AMI/IHD: acute myocardial infarction/ischemic heart disease; CMR: cardiovascular magnetic resonance; CRP: c-reactive protein; cTnI: cardiac troponin I; NT-proBNP: N-terminal pro-B-type natriuretic peptide. Continuous variables are reported as medians [IQR]. vs. cardiomyopathy: ** *p* < 0.01 and * *p* < 0.05.

## Data Availability

The data presented in this study are available on request from the corresponding author.

## Abbreviations

AMI: acute myocardial infarction; BNP: B-type Natriuretic Peptide; CMR: cardiovascular magnetic resonance; CRP: C-reactive Protein; ESC: European Society of Cardiology; HF: heart failure; hs-Troponin I: High-sensitivity Troponin I; ICD: implantable cardioverter–defibrillator; IHD: ischemic heart disease; IQR: interquartile range; MINOCA: myocardial infarction with non-obstructive coronary arteries; NSTEMI: non-ST-elevation myocardial infarction; NT-proBNP: N-terminal pro-B-type Natriuretic Peptide.
